# Sustainable use of CRISPR/Cas in fish aquaculture: the biosafety perspective

**DOI:** 10.1007/s11248-021-00274-7

**Published:** 2021-07-25

**Authors:** Arinze S. Okoli, Torill Blix, Anne I. Myhr, Wenteng Xu, Xiaodong Xu

**Affiliations:** 1 GenØk -Centre for Biosafety, SIVA Innovation Centre, Tromsø, Norway; 2 Yellow Sea Fisheries Research Institute, Chinese Academy of Fishery Sciences, Qingdao, 266071 China; 3 Qingdao Vland Biotech Company Group, Qingdao, 266061 China; 4 The Norwegian College of Fishery Science, The Arctic University of Norway (UiT), Tromsø, Norway

**Keywords:** CRISPR/Cas, Aquaculture, Salmon, Risk assessment, Sustainability, Genome-editing, Gene modification, Genetically modified organism, GMO

## Abstract

Aquaculture is becoming the primary source of seafood for human diets, and farmed fish aquaculture is one of its fastest growing sectors. The industry currently faces several challenges including infectious and parasitic diseases, reduced viability, fertility reduction, slow growth, escapee fish and environmental pollution. The commercialization of the growth-enhanced AquAdvantage salmon and the CRISPR/Cas9-developed tilapia (*Oreochromis niloticus*) proffers genetic engineering and genome editing tools, e.g. CRISPR/Cas, as potential solutions to these challenges. Future traits being developed in different fish species include disease resistance, sterility, and enhanced growth. Despite these notable advances, off-target effect and non-clarification of trait-related genes among other technical challenges hinder full realization of CRISPR/Cas potentials in fish breeding. In addition, current regulatory and risk assessment frameworks are not fit-for purpose regarding the challenges of CRISPR/Cas notwithstanding that public and regulatory acceptance are key to commercialization of products of the new technology. In this study, we discuss how CRISPR/Cas can be used to overcome some of these limitations focusing on diseases and environmental release in farmed fish aquaculture. We further present technical limitations, regulatory and risk assessment challenges of the use of CRISPR/Cas, and proffer research strategies that will provide much-needed data for regulatory decisions, risk assessments, increased public awareness and sustainable applications of CRISPR/Cas in fish aquaculture with emphasis on Atlantic salmon (*Salmo salar*) breeding.

## Introduction

### Trends in application of CRISPR/Cas in fish aquaculture

Aquaculture industries worldwide are experiencing pressing challenges including infectious and parasitic diseases, reduced viability, fertility reduction, slow growth, escapee fish, environmental pollution, coastal conflicts, and disputes regarding patenting of research outputs (Ahmed et al. 2019; Gratacap et al. 2019b). The commercialization of the growth-enhanced AquAdvantage salmon (AAS) for food in 2016 in Canada and 2019 in USA (Sweet 2019), and Nile tilapia (*Oreochromis niloticus)* in 2018 in Argentina (Evans 2018) showed that genetic engineering can proffer solutions to some of these challenges. The AAS was produced using a classical gene modification (GM) technique whereby an Atlantic salmon (*Salmo salar*) egg was modified with a gene construct containing Chinook salmon (C. Salmon) (*Oncorhynchus tshawytscha*) growth hormone gene placed under the anti-freeze protein promoter of an Ocean pout (*Macrozoarces americanus*) (Leggatt 2013). The tilapia was modified using the more recent genome editing (GE) technique, CRISPR/Cas9 (clustered regularly interspaced short palindromic repeats/CRISPR-associated protein 9), but information on the exact modification is presently not publicly available (Evans 2018). The advent of these more efficient and cheaper GE techniques, especially CRISPR/Cas, has led to GE being proposed as a potential solution to several of the current challenges of the aquaculture industry. The most targeted traits (Blix et al. 2021) for GE in fish aquaculture are reproduction and development (eg. Jin et al. 2020; Straume et al. 2021), growth (eg. Sun et al. 2020), pigmentation (eg. Xu et al. 2019; Chen et al. 2019), disease resistance (eg. Kim et al. 2021), use of trans-GFP in research (eg. Gratacap et al. 2020) and omega-3 metabolism (eg. Datsomor et al. 2019a, b).

In several studies CRISPR/Cas9 have been used to edit different genes in Atlantic salmon (Datsomor et al. 2019a, b; Edvardsen et al. 2014; Güralp et al. 2020; Straume et al. 2020, 2021; Wargelius et al. 2016) aimed at mitigating some of the problems of salmon aquaculture. For example, CRISPR/Cas9 has been used to develop a sterile salmon through knockout of the dead end (*dnd)* gene*;* the aim being to prevent hybridization and potential gene introgression of escapee farmed salmon into wild populations (Güralp et al. 2020; Wargelius et al. 2016). The technique has also been used to understand the role of the *elov-2* gene in omega-3 production of Atlantic salmon (Datsomor et al. 2019b). Other examples include use of CRISPR/Cas9 in immunological studies on different salmon species (Dehler et al. 2016, 2019; Gratacap et al. 2019b) and as a research tool (Dehler et al. 2016, 2019; Gratacap et al. 2019a, b; Chen et al. 2018; Cleveland et al. 2018).

Apart from the CRISPR/Cas9-modified tilapia, CRISPR/Cas9 is being used to modify several other traits in different species (Gratacap et al. 2019b; Zhu and Ge 2018; Blix et al. 2021). The most widely edited species are Nile tilapia, Zebra fish (*Danio rerio*) and Medaka (*Orizyas latipes*) (see Reviews by Gratacap et al. 2019b; Zhu and Ge 2018; Blix et al. 2021). Specific reproduction traits targeted for editing in Nile tilapia are sterility (eg. Jin et al. 2020), fertility (eg. Chen et al. 2017; Yan et al. 2019) and sex determination (eg. Li et al. 2015; Liu et al. 2020). In addition, editing of non-coding sequences to induce deletion of large fragments of microRNA and 3´untranslated regions (3´UTRs) has been conducted in tilapia (Li et al. 2019); this is one out of few studies using finfish species to attempt homology directed repair (HDR). Others are in Atlantic salmon targeting pigmentation (Straume et al. 2020) and sterility (Straume et al. 2021); and in farmed carp *(Labeo rohita*) (Chakrapani et al. 2016) and channel catfish (*Ictalurus punctatus*) (Elaswad et al. 2018a, b) targeting disease resistance and insertion of transgenes (Simora et al. 2020).

Further application of CRISPR/Cas in other fish species of commercial importance (for foods and ornamental value) include editing of disease resistance genes in grass carp *(Ctenopharyngdon idella*) (Ma et al. 2018), farmed carp (Chakrapani et al. 2016), and channel catfish (Elaswad and Dunham 2017; Elaswad et al. 2018a, b). Editing of growth-related genes has been conducted in common carp (Zhong et al. 2016), channel catfish (Khalil et al. 2017), tiger puffer fish (*Takifugu rubripes*), red sea bream (*Pagrus major*) (Kishimoto et al. 2018, 2019) and in olive flounder (*Paralichthys olivaceus*) (eg. Kim et al. 2019). In addition, studies in different species targeting pigmentation as a commercial trait as well as a visual tracer for research purposes have been reported (eg. Chen et al. 2019; Edvardsen et al. 2014; Liu et al. 2019; Mandal et al. 2020; Wargelius et al. 2016; Xu et al. 2019). In this study we focus on fish of commercial importance with special emphasis on salmon.

Despite the notable advances in application of CRISPR/Cas in fish aquaculture, risk assessment (RA) and regulatory approval as well as public and consumer acceptance are key to commercialization of the products of CRISPR (and other GE) technology. Apart from the two commercialized fish—the transgenic AAS and the GE tilapia, several others are at advanced stages of development, but regulatory and RA frameworks as well as requisite data and experience for evaluating the safety of these products are lacking. Consumer confidence and public acceptance of the new technology are predicated on ability of respective authorities to demonstrate robust, transparent and trustworthy regulatory and RA oversights. Several authorities have begun revising their frameworks to bring them in tandem with the envisaged challenges that will necessarily arise from GE products. These have mainly focused on plant GE products (Eckerstorfer et al. 2019b) with the result that revision and update of frameworks for GE fish and other aquacultural products lags (Larson et al. 2013; European Commission/SWD 2021). Here we highlight some of the most important issues that bedevil use of existing regulatory and RA frameworks for GE fish with focus on CRISPR/Cas-edited fish, and suggest research strategies that can ameliorate these. We also discuss some of the important technical challenges as well as pertinent issues surrounding sustainability and public acceptance of the technology in fish aquaculture.

### Genome editing (GE) techniques

In the current political debate and regulatory literature (and in this study), the term GE (also termed new genetic modification -nGM) is used to denote the emerging molecular biology techniques that make targeted (inserting, deleting or substituting) changes to an organism´s DNA. The relatively older non-targeted molecular biology tools for genetic modification are termed GM techniques (classical or old GM techniques are also used in the literature). Techniques that belong to GE are CRISPR/Cas [including all the variants that are being developed; the most advanced being the CRISPR/Cas9 variant (Larson et al. 2013; McDonald et al. 2016; Qi et al. 2013)], zinc finger nuclease (ZFN), transcription activator-like effector nuclease (TALEN), oligonucleotide directed mutagenesis (ODM), and meganucleases. These GE techniques are termed site-directed nucleases (SDN) because, unlike the old GM techniques, SDNs are directed to a specific part of the genome where they induce targeted and precise mutations (EFSA 2012). The ODM and meganuclease (meganuclease is also an SDN, but is relatively very cumbersome to use (Silva et al. 2011)) have been phased out by the less cumbersome SDNs comprising CRISPR/Cas, ZFN and TALEN, of which CRISPR/Cas is the most popular. The European Food Saftey Authourity (EFSA) (EFSA 2012) has defined three categories of SDNs viz: SDN-1, SDN-2 and SDN-3. In SDN-1, only SDNs are stably or transiently introduced aimed at generating random mutations at the target site, thus, repair of damaged host DNA is by endogenous nucleotides. SDN-2 uses small non-protein coding homologous repair DNA (donor DNA) to achieve specific nucleotide sequence changes by HDR. In SDN-3 a large stretch of protein coding donor DNA (up to several kilobases) is targeted for insertion, also by HDR, at a predefined genomic locus (EFSA 2012).

Presently, CRISPR/Cas is the most popular SDN because of its relative ease of use, low cost and high efficiency (Wang et al. 2016). The most advanced variant of CRISPR/Cas, CRISPR/Cas9, makes double-strand cuts at specific target sites on the DNA inducing a repair of the cut sites by the cell´s endogenous DNA repair mechanisms: HDR and non-homologous end joining (NHEJ) (Jiang and Doudna 2017). The NHEJ is error prone, thus the repair process often leads to alteration of the DNA sequence in the form of deletion, insertion or substitution of nucleotides (Jiang and Doudna 2017). Such alterations can render the target gene non-functional, i.e., knocked out (KO), which is desirable in gene knockout applications. Exogenous DNA sequences can be used as templates via the HDR repair mechanism to introduce donor nucleotide sequences through substitution and insertions at target sites (Jiang and Doudna 2017). Although HDR is not the prefered DNA repair mechanism, simultaneous addition of homologous DNA sequences during the DNA repair of double strand breaks (DSBs) can shift the balance from NHEJ to HDR (Jiang and Doudna 2017). The introduction at the target site of a single strand break rather than a DSB can also shift the balance in favor of HDR (Jiang and Doudna 2017). Thus, these strategies are being used to make undirected nucleotide changes (SDN-1) or directed nucleotide changes (SDN-2 and SDN-3) at targeted gene sites. The SDN-3 differs from SDN-2 in that the former leads to the insertion of protein coding transgene(s) while the latter inserts small non-protein coding sequences, e.g., regulatory sequences (EFSA 2012).

Changes in the genetic material of an organism can give rise to perturbations in the well-orchestrated gene expression both in the vicinity of the change or at loci distant from the target sites. In theory, SDN-3, which creates a transgenic organism can lead to greater irregularities in the genome compared to SDN-1 and SDN-2 (Agapito-Tenfen et al. 2018; EFSA 2012). This has resulted in some regulatory authorities to propose less stringent pathways to the RA of products arising from SDN-1 and SDN-2, especially for SDN-1 involving small nucleotide changes, e.g. a point mutation, that can also be achieved in nature (European Commission/SWD 2021). Nonetheless, it is pertinent to ascertain that all GE products are safe for the environment and/or as food/feed before approval for commercialization, although the RA challenges of the technique will depend on type of modification, i.e., whether SDN-1, SDN-2 or SDN-3.

## Limitations in the use of CRISPR/Cas in fish aquaculture

### Technical challenges

The advantages of CRISPR/Cas notwithstanding, realization of the technique´s full potentials in fish aquaculture is hindered by some technical challenges which are summarized from both the genetic and the application perspectives:

#### Genetic perspective

1. Aquatic genomic resource is still limited, although the most important aquatic species have been sequenced (Wargelius 2019). Genome editing requires clear and robust knowledge of genetic background, in practical terms, genomic sequences. Owing to the development of sequencing technology and declining sequencing cost, the genomes of over 70 aquatic fish species have been deciphered since fugu—the first sequenced aquatic species in 2002 (Aparicio et al. 2002), which is a substantial achievement during the past decades. However, they are still too few compared with the total number of aquaculture species, which according to FAO, is over 600 (FAO 2020). Moreover and for non-model species such as the Atlantic salmon, the sequenced genomes are poorly annotated (Sundaram et al. 2017), thus, CRISPR/Cas application in aquaculture will benefit from further refinement, e.g. removal of duplications in annotations from the available genomic sequences.
2. Trait-related genes need clarification. Since genetic dissection in aquatic organisms lags behind those of human and plants, trait-related genes need to be determined. In other word, which gene should be targeted? The process of identification of target genes which is via quantitative trait locus (QTL) mapping or marker assistance, is usually a long process. Although resequencing technology now facilitates the process, identification and confirmation of polygenic determined trait still hinders precise identification of candidate genes.
3. Duplication event in fish. Amongst aquatic organisms, fish represents the category with the most abundant species. However, teleost experienced a teleost-specific whole genome duplication (TS-WGD) (Glasauer and Neuhauss 2014). In salmon this issue is expanded with the salmon-specific 4th round (Ss4R) (Glasauer and Neuhauss 2014). The manner in which the duplication hinders the editing efficiency of GE techniques in, e.g., finfish has been discussed (Chen et al. 2018; Cleveland et al. 2018; Datsomor et al. 2019b; Gratacap et al. 2019a), and comparison between genes with various copies in the genome could be performed to elucidate this issue.

#### Application perspective

1. Egg membrane makes success rate of microinjection low for oviparous fish. For ovoviviparous fishes, there is no established gene editing platform at present.
2. The detection of off-target effect in model organisms focuses more on knockout efficiency (i.e. via the NHEJ/SDN-1 approach) in order to optimize CRISPR/Cas design. While as food resource, off-target effect in aquatic organisms should also focus on the impact of addition of new genes through transgenesis or cisgenesis (i.e. via HDR/SDN-3 approach). This requires more careful assessment, including both off-target in the genome and potential risk related to food quality or safety. Options for prevention or detection of off-target mutations are careful design of the annealing gRNA by comparison to existing genome assemblies, or by screening for unexpected mutations post-editing. Regarding the latter, natural genetic variation in different families and strains leaves detection post-editing complicated (Blix et al. 2021).
3. No standard protocol exists due to various features of aquatic organisms, which requires species-specific design such as needle type, injection dosage, etc. Due to lack of established cell lines and small size of egg and embryo in crustacea and molluscs, successful GE has been reported only in *Crepidula fornicate*, *Exopalaemon carinicauda,* and *Crassostrea gigas* (Gui et al. 2016; Perry and Henry 2015; Yu et al. 2019).

### Others

1. In many aquatic species, the generation interval is rather long, which makes the acquisition of mutated homozygous individuals rather time-consuming during GE process. However, it is a possible solution to combine GE with surrogacy technology (Jin et al. 2021).
2. Sterile organism is especilly favored during commercial application due to these two reasons: protection of intellectual property and the avoidance of GE individuals' invasion into wild population. However, this would require increased effort at keeping heterozygous individuals for population maintenance. Recently, Güralp and colleagues (Güralp et al. 2020) reported a method that could rescue the germ cell in *dnd* crispant-embryos of Atlantic salmon, which could then produce sterile offspring (germ-cell free) through the genetically sterile broodstock.

### Possible solutions to these challenges

#### Genetic perspective

1. The decreasing cost of sequencing (less than $10/sample) will see more aquatic genomes being deciphered in future, which will lay the necessary genomic foundation for future GE events.
2. Increasing refinements in QTL and genetic and molecular biology methods (e.g. QTL mapping, comparative genomics, and pooled CRISPR screens) will result in more trait-related genes being identified (details reviewed by Houston et al. 2020). On the other hand, specific genes that confer favourable traits across species and lines should be focused as promising candidates. For example, the current study (e.g. https://www.fhf.no/prosjekter/prosjektbasen/901631/) on the transfer of resistance to sea lice from Pacific salmon species to Atlantic salmon (Barrett et al. 2020) might result in de novo idenfication of resistant genes.
3. In terms of the trait involved in several genes (quantitative trait), generating multi-gene knockout mutants simultaneously by CRISPR/Cas will provide the possibility of inducing the desired phenotype.

*Technically* great success has been achieved in some fish species with obtaining various GE lines, especially Atlantic salmon and tilapia. These species should be employed as “aquaculture models” to initiate the optimization of aquatic CRISPR/Cas protocols and physiological assessment of potential off-target effects (for food quality and safety). The derivable knowledge from this approach is transferable to other fish species.

## How suitable are the current regulatory and risk assessment frameworks for challenges arising from CRISPR-modified GE fish?

### Regulatory and risk assessment frameworks

Many countries have developed regulatory frameworks to guide approval for environmental release and/or use of GM organisms as food, feed and fiber (Ishii and Araki 2017; Turnbull et al. 2021). The main element in these regulatory frameworks is a mandatory RA of human safety and environmental risks. The regulatory trigger is based on how a GMO is defined, which has some differences among different regulatory authorities, whereby some focus on the process by which the product is modified while others focus on the novelty of the final product (Eckerstorfer et al. 2019b; Turnbull et al. 2021). In the EU´s Directive 2001/18/EC (EU-Directive 2001), a GMO is defined as “the genetic material of the organism has been altered in a way that does not occur naturally by mating and/or natural recombination”. The Directive mandates that all GM animals and crops be subjected to regulatory review via stipulated detailed procedures. The European Commission (EC) is the regulatory authority in the EU, although at the Pan-European-level, each member country has respective regulatory body that liaises with the EC.

Different regulatory authorities throughout the world including the EU, Argentina, Brazil, Australia, New Zealand, Canada, USA and Norway have begun discussions on how to regulate products arising from the new GE techniques (Eckerstorfer et al. 2019b; Turnbull et al. 2021). Overviews of regulatory frameworks have been published by Ishii and Araki, 2017 and Turnbull et al. 2021. Box 1 provides the present state of discussions in Norway, China and the United Nation´s Convention on Biodiversity. China is at present the leading country on publications on GE in fish aquaculture, while the most publications on GE of salmon is from Norway (Blix et al., 2021).

**Box 1:** Examples of GMO regulatory frameworks

*GMO regulation in China*	
In China, the testing, production and marketing of GMOs are subject to government approval. The regulation of GMOs is primarily provided by the agricultural GMO regulations enacted by the State Council in 2001 and relevant administrative rules. Agricultural GMO regulations regulate not only crops, but also animals, microorganisms and products derived from these sources. Foreign companies that export GMOs, including GMOs as raw materials, to the People Republic of China, must apply to the Ministry of Agriculture and obtain GMO Safety Certificates – see English translation at: https://www.loc.gov/law/help/restrictions-on-gmos/china.php and https://link.springer.com/chapter/10.1007/978-981-13-8102-7_15	
At the moment, there is no separate regulation of products of GE in China since it is still under debate whether products of GE techniques belong to GM category, but the general rules for GM organism applies, which can be summarized as `ensuring safety, independent innovation, active research and careful promotion`. GM soybean and cotton have been imported and widely cultivated in China. Nevertheless, indigenous developed GM crop is limited, although safety certifications of three major GM crops (two rice and one maize variety) were approved by Ministry of Agriculture and Rural Affairs since 2014 and renewed in 2019 (valid for five-year duration) (Ministry of Agriculture and Rural Affairs 2019)	
*Norway*	
The Norwegian Gene Technology Act (NGTA) of 1993 (Norwegian Gene Technology Act 1993) requires consideration of health and environmental safety, ethical aspects, social utility and contribution to sustainability of GMOs. The first paragraph states: “The purpose of this Act is to ensure that the production and use of genetically modified organisms and the production of cloned animals take place in an ethically justifiable and socially acceptable manner, in accordance with the principle of sustainable development and without adverse effects on health and the environment” (NGTA 1993 §1) (Norwegian Gene Technology Act 1993). More specifically, the Act lays down that GMOs may only be approved when there is no risk of adverse effects on human or animal health or the environment, and that “considerable weight shall be given to whether the deliberate release will be of benefit to society and is likely to promote sustainable development” (NGTA 1993 §10,2)	
Norway is not a part of the EU, but as a member of the European Economic Area (EEA), the EU legislations -Directive 2001/18/EC, is applicable. Consequently, an approval of a GMO in EU automatically leads to an approval in Norway, unless Norway specifically prohibits importation of the product. The Norwegian Biotechnology Advisory Board (NBAB) has suggested a relaxed regulation depending on the level of GE modification, i.e. SDN-1, SDN-2 or SDN-3. An expert committee has been appointed to elaborate on this among other issues; the final report will be published in 2022	
*CBD-CPB*	
The Cartagena Protocol on Biosafety (CPB) to the Convention on Biological Diversity (CBD) is an international agreement that aims to ensure the safe handling, transport and use of living modified organisms LMOs (LMO is used in the CP in place of GMO). The CP has been ratified by 173 countries (Cartagena Protocol 2000). The protocol adopts the precautionary principle and has an established biosafety clearing house to facilitate exchange of information. Both the EU, Norway and China have ratified the protocol, while some of the major producers of GMOs including the USA, Argentina, Canada have not ratified the protocol. At present, GE is discussed as a topic in synthetic biology under the CBD, and hence not directly under the CP. An expert group -the Ad Hoc Technical Expert Group (AHTEG) has been mandated to deliberate on this, and it is expected that the outcome of the deliberation will be presented in the next meeting of the parties to the CBD in the third quarter of 2021 in ChinaArgentina, Canada have not ratified the protocol. At present, GE is discussed as a topic in synthetic biology under the CBD, and hence not directly under the CP. An expert group -the Ad Hoc Technical Expert Group (AHTEG) has been mandated established to deliberate on this, and it is expected that the outcome of the deliberation will be presented in the next meeting of the parties to the CBD in the third quarter of 2021 in China	

The main issue is: should organisms modified by GE be regulated using the existing GMO regulatory frameworks? The European Court of Justice (ECJ) in its ruling of 2018 (Court of Justice of the European Union 2018) made it clear that the established EU´s exemption of mutagenesis is only relevant for organisms obtained through methods of mutagenesis that have been conventionally used in the past and have a history of safe use. The GE techniques, including CRISPR/Cas, are not covered under this exemption given that they do not yet have any history of safe use. This implies that all applications for approval of GE products will trigger the current GMO regulatory frameworks in the EU. However, this decision has been contested: while waiting for the decision of the ECJ, Sweden used its national legislation to exempt products of SDN-1 from regulation while regulating products of SDN-3 as GMOs (Eriksson 2018). Similarly, in Argentina the SDN-1 CRISPR/Cas9-modified tilapia is exempted from regulation (Evans 2018). More amendments to the regulatory frameworks are expected as better understanding and insights are obtained regarding the GE techniques.

The present regulatory discussions on GE products by various authorities (Eckerstorfer et al. 2019b; Turnbull et al. 2021) is expected to result in the revision of the existing RA guidelines. For example, Canada recently initiated the review of its RA requirements for products arising from GE techniques (Eckerstorfer et al. 2019b); the European Food Safety Authority (EFSA) has been mandated by the EU to provide an opinion on the type of risks associated with plants produced through SDN-1 and SDN-2 approaches (EFSA 2019), but has not been mandated for an opinion or revision of guidelines regarding RA of GE animals including GE fish. The need for revision of the existing RA guidelines has also been emphasized in recent study mandated by the European Commission on the status of new genomic techniques under Union law and considering the ECJ´s 2018 ruling (European Commission/SWD 2021).

At present specific RA frameworks and guidelines do not exist for organisms and products developed by GE technologies including CRISPR/Cas. Thus, for assessment of GE products, risk assessors currently adopt/tailor the frameworks originally developed for GMOs (Agapito-Tenfen et al. 2018); see Box 2 for definitions of terms and concepts used in RA. For GE fish, the current practice is to use the general RA guidelines developed for GM animals. In recognition of the problem, the United Nations (UN), through the Conference of the Parties serving as the meeting of the Parties to the Cartagena Protocol on Biosafety (COP-MOP), recently mandated a process towards developing guidance materials on RA for GE fish (Sweet 2019). However, in terms of living modified fish, the guidelines will not be adapted as the CBD has decided not to develop additional guidance materials on RA. This implies that any application needs to follow the current guidelines. The Cartagena Protocol on Biosafety (CPB) describes five main RA steps: an identification of any novel genotypic and phenotypic characteristics, an evaluation of the likelihood of adverse effects, an evaluation of the consequences should these adverse effects be realized, an estimation of the overall risk, and a recommendation as to whether or not the identified risks are acceptable or manageable (Cartagena Protocol 2000).

**Box 2:** Risk Assessment: definitions of terms & concepts

**Risk:** the likelihood of an adverse event happening, and the seriousness of the harm represented by the event´s occurrence (Raybould 2020). Risk has also been defined as ´hazard multiplied by exposure` (Dorne and Fink-Gremmels 2013). The decision maker, i.e., the regulatory authority of a country decides the level of risk allowable for a given GMO event	
**Risk assessment:** the process of determining the occurrence, frequency and consequences of harmful events	
**Hazard:** is an event or substance that can have harmful effect	
**Harm:** an event or substance that can have adverse effect on the goals that the regulatory authority wishes to protect, such as wild species, biodiversity, human and animal health, etc. For an illustration of pathway to harm, see (Raybould 2020)	
**Aims of risk assessments**: (i) *Environmental risk assessment (ERA)*: aims to identify potential impacts on the valued components (protection goals) of the environment, and to estimate the probability and magnitude of these impacts if a GMO is accidentally or intentionally introduced into the environment	
(ii) *Food safety risk assessment:* aims to identify substances in the GMO that may be hazardous (such as toxicity or allergenicity) to human or animal health	
**Risk assessment methodologies:**	
*(a) Based on statistical nature of output:*	
(i) Qualitative: produces nominal (e.g. list of endangered species) or ordinal (e.g. low, medium, high) outputs;	
(ii) Semi-quantitative: produces interval variables (e.g. 1–5, 5–50, > 50) as outputs;	
(iii) Quantitative: produces continuous risk estimates, which may or may not be grouped into categories	
*(b) Based on period of occurrence of event:*	
(i) Retrospective: attempts to identify the causes and characteristics of harmful events that have already occurred;	
(ii) Predictive: seeks to predict the likelihood and consequence of a harmful effect that has not yet occurred. See (Kapuscinski 2007) for a comprehensive discussion on types of risk assessment	

### Challenges and limitations of the current RA guidelines

Apart from the CRISPR/Cas9-edited tilapia, there are no other commercial GE fish species, therefore, experience as well as guidance for specific RA of fish modified by GE techniques, including CRISPR do not exist (CBD/SBSTTA 2020; Sweet 2019). Even for plants (and livestock to a lesser extent) which have greater number of commercialized CRISPR/Cas-edited products, there exists no specific and harmonized guidelines tailored for their RA, such that in the EU for example, the current GMO guidelines, which recognizes all GE products as GMOs are used (EFSA 2020). The situation is the same in Canada, the USA and China which are some of the leading countries in the production and export of both GE and GM products (Turnbull et al. 2021). Several of the aquaculture end products (including fish) of CRISPR/Cas [for fish and aquaculture products currently being developed using CRISPR/Cas technique, see reviews and book chapter by (Dunham and Su 2020; Gratacap et al. 2019b; Wargelius 2019)] will at some point be evaluated for commercialization. This underscores the need to evaluate the suitability of the existing RA frameworks. The existing RA frameworks, which are tailored for products of the classical GM techniques are not adequate for products arising from the new GE techniques, given that RA issues related to the latter are different (Benessia 2015; Dunham and Su 2020; Eckerstorfer et al. 2019a; Kawall 2020; Lema 2021; Turnbull et al. 2021). The main difference is that GE technologies, in particular CRISPR/Cas, has the potential for numerous new genetic possibilities due to its efficiency, robustness and ease (Agapito-Tenfen et al. 2018; Eckerstorfer et al. 2019a; Kawall 2019), and can make greater genetic intrusions with farther reaching consequences. Further, there is insufficient prior knowledge on new traits being developed in fish and other aquaculture products using CRISPR/Cas, many of which may be difficult (or impossible) to derive comparable information on their activities from non-modified near isogenic comparators. Added to these is that unintended effects of CRISPR/Cas technology are essentially different from those of the GM techniques. Some of the unintended effects associated with the CRISPR/Cas technology, which are relevant for RA of GE fish include: unintended changes at genome locations different from the target site, i.e., off-target mutations; unintended changes at the target site associated with the specific CRISPR/Cas modification process (i.e. unintended on-target site mutations) (Kosicki et al. 2018). Similar to the effects of GM in plants, these unintended effects can lead to undesired pleiotropic effects such as abnormal expression of endogenous genes due to integration of non-endogenous sequences in sites not intended for modification (Ladics et al. 2015; Latham et al. 2006; SAM 2017). Undesired pleiotropic effects can potentially also occur even with perfectly targeted editing of the desired gene, which underpins the need for phenotypic comparison of edited product with the non-edited isogenic counterpart during RA. Further, integration of vector backbone into cells (Braatz et al. 2017); effects of methods used to facilitate uptake of the genetic molecule (Mehrotra and Goyal 2012) (Cas/sgRNA in the case of CRISPR/Cas) such as microinjection, electroporation and lipofection; effects of specific RNA or ribonucleoprotein complexes (Latham et al. 2006); have all been shown to cause abnormal expressions of endogenous genes. The impacts of these on both environmental risk assessment (ERA) and RA for food safety, as elaborated below, is varied and depends on the GE fish species, respective trait, type of modification (SDN-1, SDN-2 or SDN-3) and the receiving environment.

#### ERA

The cardinal ERA issues of GE fish are related to release (both intentional and inadvertent) in the environment because such escapees can; a) hybridize with the wild population and can, for SDN-3, lead to dispersion of transgenes (Devlin et al. 2010; Oke et al. 2013; Wringe et al. 2018), and/or b) interfere with existing biodiversity. The environmental risks associated with escapees are (a) changes to the population genetics of closely related species in the receiving environment via mating and alteration to genetic biodiversity (Hayes 2007; Kapuscinski 2007); (b) disturbance in ecological balance via alteration of the food web and destruction of habitat. However, this pertains less to GE fish in inland and contained aquaculture facilities, and more to GE fish in aquaculture systems located in waterways or marine/coastal environments where escapee farmed fish and inadvertent introduction of farmed fish into the environment are possible. Physical and biocontainment barriers are mandatory conditions for ERA of GE fish (Devos et al. 2019; Kapuscinski 2007), nevertheless, these two interventions are not guaranteed. For example, the biocontainment strategy through polyploidy (the induction of 3 or more chromosomes in fish eggs to reduce their fertility), which was employed as part of the transgenesis of the commercialized AAS (Devlin et al. 2010) is leaky because a small percentage remains as diploid fertile fish expressing growth hormone, such that there could be some fertile individuals among escapee salmon (Benfey and Sutterlin 1984). Besides, generating polyploidy in fish is also an animal welfare issue. Similarly, physical containment is not foolproof because escapee farmed Atlantic salmon have been reported at wild salmon spawning grounds (Bergan et al. 1991; Gausen and Moen 1991; Jensen et al. 2013). This has led to criticism on the extent of the physical and biocontainment conditions of the ERA that was conducted for the commercialized AAS (Benessia 2015; Sweet 2019) especially given the difficulty of providing or predicting environmental impacts of release of GE fish. Unfortunately, no information on ERA is publicly available for the commercialized GE tilapia. The challenge to ERA is whether the effects of hybridization and transgene introgression of the released GE fish can affect overall fitness including survival, migration, spawning, reproduction, etc., of the wild population. The sterile GE salmon by the Wargelius group (Wargelius et al. 2016; Güralp et al. 2020), as a proof of prinicple, can prevent hybridization from SDN-1 and SDN-2 fish, and transgene introgression from SDN-3 fish via interbeeding between a released GE fish and wild population, but the impacts of such modification on overall fitness of the fish under natural conditions have not been conducted. Unfortunately, the natural and environmental conditions that influence these factors cannot be studied under controlled laboratory conditions (Leggatt et al. 2017; Sundstrom et al. 2007). However, the concept of combining sterility trait and other traits in the same GE fish increases the prospect of avoiding hybridization with wild relatives and controlling transgene introgression by escapee GE fish from open water commercial fish farms.

#### RA for food safety

The aim of RA for food safety is to ensure that the process of modification as well as the effects (both intended and unintended) have not resulted in toxicity, allergenicity and/or decreased food quality (i.e. undesirable biochemical composition of the edible tissues) of the modified fish (EFSA 2013). Effects of the CRISPR/Cas modification process, unintended effects as well as intended new traits can affect the fitness and hence the quality of the actual edited fish. Currently, systematic studies have not been conducted on the impact of unintended effects of GM (or CRISPR/Cas) on the quality/safety of edible tissues of GE fish (or any GM or GE modified animal), although unintended changes have been reported to alter disease resistance, foraging behavior, gene expression, reproduction and life-history timing (Abrahams and Sutterlin 1999; Devlin et al. 2015). Extrapolations from specific examples of past experiences with GM and genetic engineering technologies can help deduce potential impacts of CRISPR/Cas on the quality of edited fish for RA purposes. For example, biochemical analyses of the components (carbohydrates, proteins, total fats, vitamins and minerals) of the edible tissues of the AAS show no significant variations compared with the unmodified counterpart, except a slight variation in the concentration of vitamin B6 (Benessia 2015). The mRNA expression levels of the following proteins were reported as increased in a growth-modified transgenic amago salmon (*Oncorhynchus masou*): haeme oxygenase; leukocyte cell-derived chemotaxin; α-trypsin inhibitors; iron metabolic proteins; and proteins of the reproductive system, while the expressions of lectin, D-6-desaturase, apolipoprotein and pentraxin were reduced (Mori et al. 2007). In a transgenic coho salmon, glutathione levels; glutathione reductase and gamma-glutamiltranspeptidase activities were increased by growth hormone modification (Leggatt et al. 2007). A CRISPR/Cas9-based ablation of *elovl2* gene (an essential gene in synthesis of long chain polyunsaturated fatty acids LC-PUFA), resulted in the accumulation of different polyunsaturated fatty acids, and up-regulation of several genes involved in fatty acid metabolism in Atlantic salmon (Datsomor et al. 2019b). The extent to which these can impact the quality of edible tissues of the GE fish is unknown. The AAS was also evaluated for allergenicity via dermal contact (Leggatt 2013) according to present RA standard procedures. However, the impact of the intended increased growth hormone on the edible tissues and its possible health implications was not conducted, neither were the direct and indirect impacts on the entire cellular metabolic network performed (Benessia 2015; Van Eenennaam 2011). It is questionable whether the current standard molecular characterization, toxicity and allergenic studies for characterization of GM food safety is sufficient for products of CRISPR/Cas technology. It has been argued (Abrahams and Sutterlin 1999; Devlin et al. 2015) that GM products should also be evaluated for anti-nutrients or lowered nutrients, this may also be relevant for GE products. Others (Kawall 2020; Agapito-Tenfen et al. 2018) have also argued that in addition to standard molecular characterization, omics evaluation of GE products, especially with regard to unintended changes, will provide relevant additional data for robust RAs. Nonetheless, it is clear that determining whether these unintended changes have (or are associated) with any harmful effects is complex and presents a challenge to the present RA frameworks.

#### Issue of sustainability

Most GMO regulatory frameworks mainly include safety questions on guidelines for health and ERA. In addition to this, it is important, in the case of GE, to acknowledge the social dimensions of how natural sciences use nature (Palsson et al. 2013). Food has an impact on human lives that is of both cultural and biological importance, and it is therefore not sufficient to consider only measurable risk. Food is also about traditions and ways of life (Myskja and Myhr 2020). Several questions of risk, e.g., future effects of horizontal gene transfer, are not possible to answer in present terms. These become questions about what human changing nature might lead to, and whether this is something that society is willing to accept. Hence, the “[…] blurry line between risk and sustainability demonstrates the significance of including non-safety issues in order to make a decision that is socially acceptable” (Myskja and Myhr 2020).

There is no common practice of evaluating non-safety criteria of GMOs, even though several countries have implemented such measures (Myskja and Myhr 2020). In Norway, GMO regulation frameworks include both environmental/ecological, societal and economic dimensions, through including non-safety criteria—contribution to sustainable development, ethical justifiability and societal utility in the evaluation process of GMOs (Box 1). The criterion of contribution to sustainable development is interesting considering the frequent use of this term, e.g. in global and national aquaculture strategies and reports. However, a framework operationalizing contribution to sustainable development at present has only been developed for GM plants (NBAB 2009, 2011, 2014). An alternative to developing a framework is to make requirements for certifications under international certification schemes; an example for GE fish could be certification under the Aquaculture Stewardship Council (ASC).

#### Consumer perception and acceptability

The new possibilities of GE, especially CRISPR/Cas, as stated above, call for updated regulatory and RA frameworks and guidelines. Likewise, they demand a new public discussion on the application of the technology in food production amongst other areas of use. Historically, GM has been associated with controversy. The aspects raised in public discourses are connected to animal moral status, the argument of whether genetic engineering is natural or unnatural, the percieved risks and benefits of genetically engineered animals to health and the environment, the purpose of the application, the methods being used and the motivation of the researchers. And finally the species itself—its intrinsic value, species boundaries and animal ethics (Van Eenennam and Young 2018). These aspects may be important for public and market acceptance of GE products.

## Scientific knowledge and research-needs on risk assessment, sustainability and consumer perception of CRISPR-modified GE fish

### Available studies

The data requirements for RA of CRISPR/Cas-modified GE fish, while being similar to fish modified by other GM techniques, differ with respect to the unique process of CRISPR/Cas modification. Obtaining data relevant for RA of CRISPR-modified GE fish is at its infancy: no information is publicly available even for the only commercialized CRISPR/Cas9-modified GE fish -the GE tilapia. However, some information exist on ERA, RA of food safety, sustainability and public/consumer perception for GM fish which can be extrapolated, albeit only to a certain degree, to GE fish.

#### ERA

Some ERA-relevant studies on transgenic fish have been conducted but these relied mainly on laboratory/confined field (Devlin 2007) and modelling (Ahrens and Devlin 2011; Li 2014) studies and not on field studies. In the laboratory/confined field studies, the approach is the application of semi-natural conditions and use of surrogate models in nature (Devlin et al. 2006). EcoPath and Ecosim modelling have been applied in predicting effects of releases of growth hormone transgenic salmon (Ahrens and Devlin 2011; Li 2014). For example, computer-based modelling simulations found that presence of transgene can potentially shift genetic backgrounds and phenotypes of both GM and non-GM fish away from the naturally selected optima (Ahrens and Devlin 2011). However, it is not practicable to obtain data that accurately depict pathway to harm of GM fish when released into natural conditions (Devlin et al. 2015; Sundstrom et al. 2007). This has raised uncertainties as to the extent data generated from these studies can be used in ERA, such as uncertainties related to extrapolating results from confined tests to natural ecosystems, pleiotropic effects and phenotypic trade-offs between traits and genotype-by-environment interactions (Benessia 2015; Devlin 2007).

#### RA of food safety

There is at present no systematic data available on the pysiopathology of GE fish. Even for the most studied Salmonidae subfamily, different methodologies and research objectives have been used. For example, in the widely studied growth hormone-expressing transgenic coho salmon, amago salmon and Atlantic salmon, few studies on biochemical alterations caused by deregulation of growth hormone expression are available (Leggatt et al. 2007; Mori et al. 2007). The most available comprehensive study is the biochemical characterization on AAS by the producer—Aquabounty (AquaBounty Technologies Inc. 2010; Benessia 2015), but no independent systematic studies on AAS have been reported in the published literature. Studies on safety of GE fish for human consumption should not be limited to the direct effect of the transgene (VMAC 2010) for SDN-3 fish, nor allergens (Van Eenennaam 2011), but also extended to the direct and indirect effects on the entire metabolic network (for SDN-1, 2 and 3). In this regard, the application of new and robust molecular biology analytical techniques such as omics (in particular proteomics and metabolomics) can be useful (Agapito-Tenfen et al. 2018; Eckerstorfer et al. 2019a; Kawall 2020). For example, a research group (Datsomor et al. 2019b) recently used lipidomic and transcriptomic analyses to characterize the impact of a CRISPR/Cas9-*elovl2* knockout event on lipid biosynthetic pathway.

#### Sustainability

Many countries have agreed on Agenda 2030 and committed to the United Nations’ 17 global sustainable development goals (SDGs) for a better future (UN 2015). The overarching goal by this commitment is to make possible dignified human life while at the same time permanently and on a sustained basis protect natural conditions of life. The three key parts of sustainability is included within the SDGs: economy, society and the environment. These could be used as guidance for what measures within sustainable development should be assessed. The scope of SDGs is wide and includes 169 specific targets (UN 2015), which are important and internationally accepted goals. Adopting these widely accepted goals could also ease defending the inclusion of non-safety issues internationally. In addition to the UN SDGs, both protection of biodiversity, ecosystems and development of sustainable food production systems are part of ´A European Green Deal´ (European Commission Grean Deal 2019). For example, if use of the AAS and the GE tilapia (as well as future GE fish products) is to be evaluated for contribution to sustainability, it would be advantageous if this evaluation aligns with the action plans of the Green Deal.

Sustainable development is globally defined as development which does not reduce the possibilities of future generations while simultaneously ensuring the possibilities of present generations (Brundtland 1987). Within such a definition, using GE for ensuring a stable and efficient aquaculture production may be accepted, if the technology meets the demands of safety and risk issues, and if it contributes to social and economc sustainability, e.g., upholding transparency in the food chain, creating work opportunities and supporting local communities. Further, the welfare of animals must be morally acceptable for it to be sustainable (Blix and Myhr 2021; Broom 2010). In the Norwegian Animal Welfare Act, all animals are stated to have intrinsic value independent of their use-value to humans (Norwegian Animal Welfare Act 2009). If intrinsic value of animals is also to be taken into consideration, then a sustainable use of GE cannot compromise the moral status of the animal (Blix and Myhr 2021). Defining sustainable development as something that has to be morally acceptable also widens the extent to which perspectives of stakeholders and the public are included in evaluation of GE foods—one of the intentions of non-safety criteria (Zetterberg and Edvardsson Björnberg 2017).

#### Public/consumer perception

In 2002, the Eurobarometer on Biotechnology showed that “[…] the majority of Europeans [did] not support GM foods” (Gaskell 2003). This attitude did not change according to the findings of the Eurobarometer of 2010 (European Commission/TNS 2010). The concerns for this group of foods were safety for health, and the issues of whether it was necessary to apply GM in place of conventional breeding. In 2019, the concern seemed to have dropped slightly—only 27% of the respondents expressed concern for GM ingredients in foods or drinks (European Commission/EFSA 2019). Predictions are for GE products to be more accepted, especially for products of SDN-1, which do not involve cis or transgenesis. The main objectives for using GE are more efficient and sustainable food production, as well as economic profitability. In spring 2020, two comprehensive surveys on public perception of gene technology in food production were conducted and published in Norway by the Norwegian Biotechnology Advisory Board (NBAB 2020), and by SIFO (Consumption Research Norway) (Bugge 2020). NBAB (N = 2016) used the term ´gene editing` and showed that the Norwegian consumers´ attitudes depend heavily on what the technology is used for. With regards to use on animals, the consumers are mainly concerned with using the technology for improving health in production animals and reducing the environmental impact of protein production industries. Increasing growth or changing visual traits like color of flesh is regarded less important amongst the consumers (NBAB 2020). In the SIFO report (N = 1066), the term GMO was used in their survey, and 47% of the respondents expressed that GMOs collided with their view of what ethical food production looks like. With regards to the possible negative effects of GMOs, the respondents were most concerned with nature and ecosystems, followed by concern for their own health (Bugge 2020).

Similar surveys have also been conducted in China. Recently Cui and Shoemaker (2018) published a nationwide study (N = 2063) on public perception of GM food where a majority of the respondents answered that they are either neutral (46.7%) or opposed (41.4%) to GM food. The study also approached linking acceptance to self-percieved knowledge about GM foods, and states that there seems to be statistical evidence for a positive connection between more knowledge and acceptance. The study also emphasised a lack of trust in the public towards authorities, but also in biologists opinion, and further calls for more effort “[…] to gain confidence, trust and support from public domain” (Cui and Shoemaker 2018). However, the issues that the respondents are most concerned with are “how to identify GM food” and they also want more information about “general scientific knowledge on GM food safety” (Cui and Shoemaker 2018). This underpins the need for dissemination of knowledge connected to GM and GE foods, and that the public seeks information which will not necessarily lead to rejecting GM foods, but rather for more informed actions.

The results from these surveys indicate that the discourse on GE foods is not unambiguous. It has been emphasized that some important issues on which public acceptance is based are—naturalness, trust, one's own health, the environment and the origin of the food products that we eat (Myskja and Myhr 2020). There is a need for upholding this discussion about the origin and type of foods we want to accept and why these are acceptable (De Graeff et al. 2019). This has also recently been brought up by the European Group on Ethics in Science and New Technologies in the report `Ethics of Genome Editing`. Regarding use of GE on animals, key identified questions are how the new technology affects the balance between the three R´s (refine, replace, reduce) in research, and in general, how it affects animal welfare as well as what the implications for biodiversity are (EGE 2021).

### Proposed research strategy for relevant data on food safety RA and regulation

**Fig. 1:**
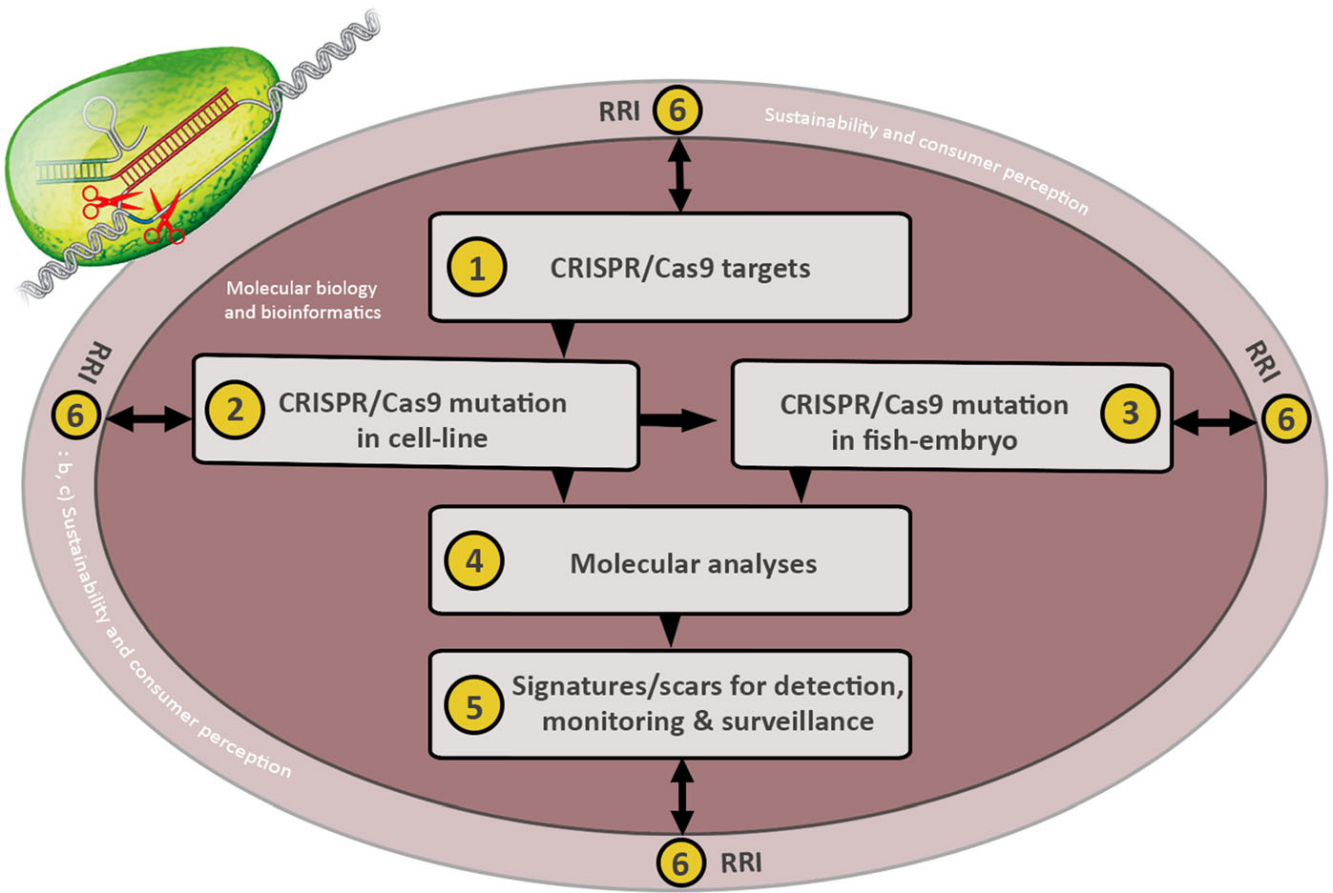
(1) identify potential CRISPR/Cas targets by a combination of large-scale genome-wide (Gratacap et al. 2019b) as well as proteome- and transcriptome-wide screening of respective in-vitro and in-vivo pathogen-challenged cells and tissues of fish; (2) identify and select high priority list of genes using a combination of CRISPR/Cas-mutations and phenotypic testing; (3) CRISPR/Cas mutations of the genes highly prioritized in (2) in fish embryo, coupled with sequencing, phenotypic testing and characterization to identify population with desired disease resistance phenotype; (4) molecular analyses of data generated in (1), (2), (3) for off-target mutations; (5) Further analyses of data for reproducible and predictable genetic changes around the target areas that are recurring and consistent, which can be used to develop genetic tools for detection, tracing and surveillance of GE fish; (6) actively integrate, at all stages of the study, key RRI aspects of purpose, process and outcome, where all actors (policy makers, research communities, business & industry, representatives from NGOs and the public) are engaged, e.g., through stakeholders participatory workshops (Agapito-Tenfen et al. 2018)

We propose a reseach strategy, which will involve the active participation of the different disciplines of molecular biology, bioinformatics (for data on unintended mutations, detection, traceability and surveillance), social sciences and humanities (for data on consumer perception and sustainability), where social sciences and humanities are encompassed in the principles of responsible research and innovation (RRI); see scheme described in Fig. 1. This will allow interaction of the different aspects of molecular biology and bioinformatics with RRI parameters, e.g. stakeholders´involvement especially with regards to identifying issues of social acceptance and public ethical justifiability in all aspects of the research. The strategy emphasizes a departure from research where the technical science and humanities/social science components of a project do not interact with each other, but instead encourage active participation and learning from each other. Data derivable from the proposed strategy will enhance transparent research and consumer knowledge about CRISPR/Cas as well as influence current global debate on application of CRISPR/Cas in production, RA and regulation of GE fish and other GE aquaculture products.

#### Unintended (off-target & on-target) mutations, detection, traceability and surveillance

Using disease resistance in GE salmon and the SDN-1 approach as examples, data on unintended off- and on-target effects as well as data on detection, traceability and surveillance can be obtained using the scheme described in Fig. 1. Gratacap et al. (2019b) have shown that combining in-vitro and in-vivo large-scale genome wide screening approach can be used to identify disease resistance alleles in aquaculture species. We further propose that studies integrating new genetic tools in omics can be used to identify recurring and consistent features of CRISPR/Cas knockout mutations in salmon and/or salmonid-derived cells using e.g. disease-related genes of Atlantic salmon, which can be relevant for detection and surveillance of GE fish. Non-random repair outcome of NHEJ of DSBs generated by CRISPR/Cas9 across cell-lines, experimental replicates and reagent delivery have been reported (van Overbeek et al 2016; Shou et al 2018). Repair outcomes were unique to each target and determined by protospaceer adjacent motif (PAM) sequence rather than genomic sequence (van Overbeek et al 2016; Shou et al 2018; Chakrabarti et al 2019), and are reproducible and predictable (van Overbeek et al 2016; Shou et al 2018; Chakrabarti et al 2019). Thus, integrated multiomics (sequencing, bioinformatics, transcriptomics, proteomics and metabolomics) can be used to profile the resulting repair-products of CRISPR/Cas9 DSB of specific genes (e.g. disease resistance genes) with the aim of identifying unique and recurring characteristics. These features can serve as unique genetic signatures or molecular markers for detection, tracing and surveillance of GE fish developed by CRISPR/Cas SDN-1 approach, providing important data for RA of food safety. This approach can also be used to identify off-target mutations and their impact on the GE fish.

#### Consumer perception

The SIFO and NBAB reports have divergent interests and methods for asking questions and have thus interpreted the answers somewhat differently. This has been emphasized and criticized (Antonsen et al 2020a, b; Carson et al. 2021). The divergent results, and the criticism of the questions asked indicate that more work is needed in this area, especially for mapping consumer perception and acceptability. We would like to direct a similar reaction to the Chinese survey on public perception by Cui and Shoemaker (2018). Analysing large surveys based on a pre-determined belief that GE organisms are safe to eat and could be made safe for the environment will not give a clear understanding of the public perception. It is important not to write off negative public perception or lack of acceptability as a lack of knowledge amongst the public. The NBAB has attempted such a comparison by asking “control” questions in the survey, in order to test the respondents` level of knowledge. Some of the criticisms in (Antonsen et al 2020a, b) focused on this and how it not only degrades public perception, but also that the control questions used were poorly formulated and misleading. Acceptance of GE organisms in food production including aquaculture is not only a question of risk and physical effects, it is also about human relation to animals and nature, instrumentalization of animals, dignity and characteristics of species, and the […] increasingly imbalanced power distribution between humans and animals” (De Graeff et al. 2019). This should be further emphasized in future surveys where stakeholders´ and public acceptance of GE are studied; see Fig. 1, (6), RRI. As shown in the NBAB study, using technology for improving health in production animals and reducing the environmental impact may be considered more acceptable than other purposes.

Future surveys aiming at mapping public perception should take all this into account. A survey might not, as seen in recent studies (NBAB 2020; Bugge 2020; Cui and Shoemaker 2018) give clear answers to whether the public supports or is opposed to the use of GE animals in food production. Acceptance has several dimensions and aspects which do not necessarily lead to a clear answer for opposing or supporting the technology (Van Eenennam and Young 2018). We encourage future surveys to be aware of this and instead of asking leading questions, focus the surveys so that they are open with the aim to identify what the most pressing concerns are.

#### Sustainability

Contribution to sustainable development is one of the non-safety criteria in Norwegian Gene Technology Act, but the regulation is not yet translated into guidelines on how to evaluate GE (and GM) fishes. This could be done by looking at global goals and strategies such as UN SDGs and EU Green Deal. Further, a framework for evaluating GE animals should be based on relevant regulatory frameworks and policy documents for animal and fish welfare (Blix and Myhr 2021). In Norway the Animal Welfare Act equalizes terrestrial and aquatic animals, designating them with rights to be protected from harm, stress and strain, in addition to other intrinsic values (Norwegian Animal Welfare Act 2009). Internationally, fish has not been assigned the same status. However, the Animal World Health Organization has developed international standards for fish welfare contained in the Aquatic Code (The Aquatic Code 2019). A framework for evaluating GE fish should use the Aquatic Code, which focuses on how to avoid disease, as a guiding minimum for how welfare is to be understood with regards to fish. In addition, it should also be considered whether the intrinsic value of fish should be taken into consideration. This could be a valuable guiding principle (Trøite and Myskja 2018) for determining whether GE diminishes the integrity of animal; alternatively what kind of GE is acceptable? (Blix and Myhr 2021).

## Conclusion

The CRISPR/Cas technique is the most popular of the current GE technologies, therefore, majority of the expected GE fish products for commercialization will be products developed by CRISPR/Cas. The technique has the potential to provide far-reaching solutions to the several challenges plaquing the fish aquaculture sector. The current national and international discussions is on whether products arising from GE techniques, especially CRISPR/Cas, should be regulated. Given the enormous importance of the matter, decisions whether to regulate or not, and more importantly, how to regulate GE fish products, should be based on knowledge derived from profound scientific research. The current RA frameworks do not cover CRISPR/Cas GE fish. This will challenge the existing frameworks with regards to unintended and pleiotropic effects as well as detection, identification, tracing and monitoring of GE fish in the case of inadvertent or intentional release into the environment. The essential knowledge for crucial decisions and robust RA is not available. Research strategies that take advantage of the new molecular biology techniques, including transcriptomics, proteomics and metabolomics, which have become more advanced and cheaper, will contribute these knowledge-needs. Further, inclusion of animal welfare, ethical, societal and sustainability aspects in the policy decision process will complement risk assessment and ensure that cultural and societal interests are taken into consideration.
